# Suicide in South Asia: a scoping review

**DOI:** 10.1186/s12888-014-0358-9

**Published:** 2014-12-24

**Authors:** Mark JD Jordans, Anne Kaufman, Natassia F Brenman, Ramesh P Adhikari, Nagendra P Luitel, Wietse A Tol, Ivan Komproe

**Affiliations:** Research and Development Department, HealthNet TPO, Amsterdam, The Netherlands; Center for Global Mental Health, Institute of Psychiatry, King’s College London, London, UK; Research Department, Transcultural Psychosocial Organization (TPO) Nepal, G.P.O Box 8974/ C.P.C. Box 612, Baluwatar, Kathmandu, Nepal; Department of Mental Health, Bloomberg School of Public Health, Johns Hopkins University, Baltimore, MD USA; Faculty of Social and Behavioural Sciences, Utrecht University, Utrecht, the Netherlands

**Keywords:** Suicide, Review, South Asia, Scoping

## Abstract

**Background:**

Globally, suicide is an important cause of mortality. In low- and middle income settings, it is difficult to find unequivocal data to establish suicide rates. The objective of this review is to synthesize the reporting of suicide incidence in six south Asian countries.

**Methods:**

We conducted a scoping review combining peer-reviewed studies (PubMed, PsycINFO, EMBASE) with in-country searches for grey literature in Afghanistan, Pakistan, Sri Lanka, India, Nepal and Bangladesh. The review included mapping reported suicide rates, quality appraisals of the studies, use of definitions of suicide and means of committing suicide.

**Results:**

In total, 114 studies and reports were included in the review, including 50 peer-reviewed publications. Reported suicide rates varied widely from 0.43/100,000 to 331.0/100,000. The average suicide rate across studies was found to be high compared to the world average, however many studies were of poor quality or not representative. The majority of studies failed to explicitly define suicide (84% of the published articles and 92% of the grey literature documents). Poisoning and hanging were consistently the most common methods of committing suicide on the sub-continent.

**Conclusions:**

The reported suicide rates in South Asia are high compared to the global average, but there is a paucity of reliable data on suicide rates in South Asia. Reports are likely to diminish rather than exaggerate the magnitude of suicide rates. There is an urgent need to establish new, or evaluate existing, national suicide surveillance systems in the South Asian countries.

**Electronic supplementary material:**

The online version of this article (doi:10.1186/s12888-014-0358-9) contains supplementary material, which is available to authorized users.

## Background

According to a recent WHO global suicide report, suicides in the world amounted to just over 800,000 deaths in 2012, representing 1.5% of total mortality and about 16% of injury mortality [1,2]. The worldwide suicide rate is estimated at 11.4 per 100,000 inhabitants, similar to the average rate reported for 2008 [3], making it the 15^th^ most common cause of death worldwide. Globally, suicides account for 50% of all violent deaths in men and 71% in women [2]. Over the past decades, the locus of the problem (in terms of magnitude) is shifting from Western Europe, to Eastern Europe to Asia [3]. Indeed, a recent review of suicide in Asia demonstrates higher average suicide rates in Asia compared to high-income countries [4]. In many low- and middle income countries (LMIC), no national suicide data is available or their reliability is questioned [3,5]. Therefore, while the figures and trends described above are important, they present with some limitations, as they exclude countries that do not have mortality surveillance system in place (e.g. for South Asia, only India and Sri Lanka have been included in most of the reviews and databases), do not assess the quality and reliability of gathered data, or rely on outdated information. Especially in LMIC, actual figures may therefore be higher. There is therefore an urgent need to obtain all available data on suicide in order to most accurately gauge the seriousness of the problem, and to establish reliable systems to collect data on suicide in South Asia. Understanding the true magnitude of the problem suicide imposes on societies is of significant public health importance, as governments need data on the social and economic burden associated with suicide to drive development and implementation of prevention programs [6]. The aim of this scoping review is to provide a comprehensive understanding of existing literature reporting suicide rates, and other suicide metrics, in six south Asian countries (Afghanistan, Bangladesh, India, Nepal, Pakistan, Sri Lanka), by reviewing both published (i.e. in peer reviewed journals) and unpublished studies (i.e. reports not published in the academic literature). In addition, we aim to appraise the quality of the studies, use of definitions of suicide, and summarize the reporting on means of committing suicide. To the best of our knowledge this is the first such review on this topic in this area of the world.

## Methods

We conducted a scoping review of peer-reviewed publications and grey literature. Scoping reviews have been described as a process of mapping the existing literature [7]. The published literature was reviewed as a systematic review, and reporting was done in accordance with the PRISMA (Preferred Reporting Items for Systematic Reviews and Meta-Analyses) statement [8]. No meta-analyses were conducted. A protocol for the study was reviewed and approved by two independent committees, one with experts from South Asia, the other with experts in conducting literature reviews. We followed Arksey and O’Malley’s framework for conducting a scoping review, following these five steps: identifying the research question; identifying relevant studies; study selection; charting the data; and, collating, summarizing and reporting the results [9]. We also included in-country consultations as part of the protocol.

### Search strategy

**Figure 1:**
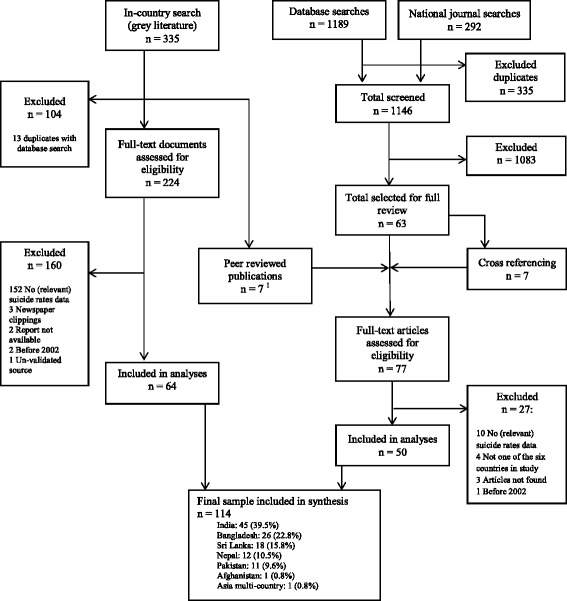
**Review flowchart.** Note: ^1^These publications refer to peer-reviewed publications that were identified through the in-country search and that did not come up in the online search of databases.

Published studies and reports were identified through a systematic search using the following strategies. First, to identify peer-reviewed publications we searched online databases (PsycINFO, PubMed, EMBASE) and key national journal databases for each country (banglajolinfo [Bangladesh], medindia.net [India], nepjol.info [Nepal], pakmedi.net [Pakistan], sljol.info [Sri Lanka] none available for Afghanistan). The search terms we used were: [suicid* AND South Asia] OR [suicid* AND Afghanistan] OR [suicid* AND Bangladesh] OR [suicid* AND India] OR [suicid* AND Nepal] OR [suicid* AND Pakistan] OR [suicid* AND Sri Lanka]. The search was performed in November 2012. Initial identification of relevant studies was based on title, keywords and abstracts. All publications that were eligible for full review were cross-referenced. Second, one consultant in each of the six countries was hired and trained to conduct a search for reports not published in the peer reviewed literature (i.e. grey literature) and other types of data on suicide rates in their respective countries. The in-country search was conducted during a six week period in the spring of 2013. These searches included online searches and face-to-face meetings with relevant representatives from government agencies such as the police department, Ministry of Health, (international-) non-governmental organizations (NGO), academic institutions and United Nations agencies, and consultation with a pool of experts generated from the literature search or recommended by members of the advisory committee (see Figure 1). All information and documents were logged and subsequently checked and validated by a member of the research team (RA).

### Inclusion and exclusion

We included articles published, or reports issued, from 2002 onward, and reporting on one of the six countries included in the study. For publications presenting data from before 2002, we only included data on suicide rates or other metrics related to suicide deaths from 1998 and later. This timeframe was chosen in order to provide a contemporary overview of suicide in South Asia. Additionally, for the peer-reviewed articles, we excluded records published in languages other than English, book chapters, conference proceedings, dissertations, editorials and commentaries. For the grey literature, no additional restrictions applied. All identified publications were initially screened based on abstract and title for relevance. This was done by two members (AK, RA) of the research team, and independently cross-checked by another member (MJ). Any questions were resolved through discussion. Subsequently, the full text of selected publications was assessed for eligibility (AK, NB, MJ). Ten percent of all full-texts reviewed was independently assessed by two researchers, any discrepancies were resolved on consensus basis. During initial screening of publications for eligibility, we included articles in which it was not clear whether it dealt with suicide attempts or suicide deaths. These articles were reviewed in full to determine whether the focus was on attempted suicides or suicide deaths, and clarify the meaning of the presented figures. In cases where this could not be determined from a reading of the full text, suicide data from the study were not included in further analyses.

### Data extraction

All records included in the data set were read again and data were entered into a pre-defined spreadsheet. This format included details on study -objectives, -period, and –methods, suicide rates or other suicide metrics (incl. gender and age differences), means of committing suicide, suicide definitions employed, and suicide reporting or registration system. All information in the spreadsheets was checked by one author (AK) for accuracy and comprehensibility. The peer-reviewed publications were appraised for quality, using a tool developed for the purpose of this study (see below). Quantitative data was entered into an SPSS file, specifically the reported suicide rates and quality appraisal score, which were used to run descriptive analyses.

### Quality appraisal

There is no clear consensus on a preferred tool for assessing the quality of observational studies, however, there are guidelines on the reporting of observational studies (i.e. the STROBE statement) [10]. Moreover, a review by Sanderson and colleagues [11] of instruments for appraising quality of observational studies recommends using tools that: include a small number of items on key domains; are as specific as possible with regard to aspects of quality that is evaluated; that are simple checklists rather than scales, given that psychometrics of scores on scales are not always substantiated; and shows evidence of careful development and psychometrics. We set out to select or adapt a tool specific to the current study, which would heed these guidelines and cover different study types. The final tool we applied in the study is a short checklist that we developed mainly based on questions developed by Boyle [12]. We adapted the questions suggested by Boyle for suicide observational studies in consistence with approaches described by the systematic reviews on suicide/ maternal death incidence [13,14].

The final tool consists of 8 items: is the target/catchment population defined clearly?; is the sampling method clearly described and adequate?; do the characteristics of respondents match the target population?; are the data collection methods standardized?; are the instruments/ways in which suicide was established reliable?; are the survey instruments/ways in which suicide was established valid?; are you confident about the authors’ choice and use of statistical methods?; are data accurately presented? Each item includes criteria for scoring. Scoring options consists of ‘yes’ (i.e. satisfied all criteria), ‘some’ (i.e. some criteria met), ‘no’ (i.e. none of the criteria met). The quality appraisals were done by two authors (MJ, NB). The inter-rater reliability between both raters was assessed on 10% of the publications on each of the criteria (n = 8), which resulted in a Cohen’s Kappa (*k*) of *k* = .67. Finally, we included an overall quality rating by adding the number of indicators fully satisfied (i.e. total of ‘yes’ scores; response range = 0-8). We have not included the middle score (somewhat satisfied) in the overall rating to err on the conservative side of the overall score (i.e. when applying such dichotomization on all scores, the level of inter-rater agreement went up to *k* = .89.

## Results

Altogether, 114 studies are identified (n = 50 peer reviewed publications; n = 64 grey literature publications from in-country searches) that meet the inclusion criteria. See Figure 1 for the study flowchart. A total of 225 organizations and/or individuals are contacted by the consultants in the six countries, and searched 190 institutional websites. (See Additional file 1 for a full overview of results.)

The range of reported suicide rates across the publications is 0.43 (Pakistan) to 331.0 (elderly people in a sub-district in India) per population of 100,000. The non-pooled mean rate for Bangladesh is 58.3 (SD = 63.22), for India it is 28.8 (SD = 32.17), for Sri Lanka it is 25.7 (SD = 4.80), for Nepal it is 8.6 (SD = 8.87), and for Pakistan it is 3.6 (SD = 5.06). For Afghanistan no publication reports actual suicide rates. The non-pooled average suicide rate across all six South Asian countries for the included time period is 25.2 (SD = 28.60). These mean rates are presented here as indications only, because they represent a crude measure with questionable reliability due to the low number of studies (e.g. Bangladesh) and large range of rates, and the fact that data from different sources or populations are combined. Consequently, we have analyzed the studies differentiating for both population and quality.

There are large differences between the rates reported for the entire population (i.e. national data) and for sub-populations (i.e. a specific province, among refugees), each representing approximately half of the included studies. Studies among sub-populations are consistently associated with higher rates than nationally representative data (mean rates of 37.55 [SD = 35.20] and 14.28 [SD = 7.60] respectively).

Results of the quality appraisals are presented in Table 1. Only one study [15] (2%) satisfied all 8 criteria and the majority satisfied four or less (80%), with seven studies scoring 0 (14%); the mean number of quality indicators with satisfied criteria was 2.6 (range 0 to 8). Most commonly, the population definition and presentation of statistics were adequate, while all the data collection and measurement techniques were not. This reflects the pragmatic use of existing data from police or medical records, which cannot be controlled or assessed for representativeness, reliability or validity. When plotting the quality of the studies against reported suicide rates, there is a trend suggesting higher reported rates for higher quality studies. Given the significant difference between national and sub-population level rates, it is essential to separate these two categories. As a strategy to compare the trends between both categories we used linear regression analyses (with ‘suicide rates’ as outcome and ‘quality of study’ as predictor) and used the estimated β as an indicator of the likelihood of the trend [16]. According to this strategy the estimation of a significant β confirms a significant trend in the plots. The regression analyses confirm this overall trend (β = .017; SE = .006; p = .012), and specifically for the sub-population studies (β = .015; SE = .005; p = .009), yet fail to do so for the national studies due to a lack of included studies (β = .100; SE = .126; p = .463). The highest quality studies (10 studies scoring 5 or more; 20%) are mainly characterized by: (a) gathering data from large representative samples of the target population or entire demographics using a health surveillance system or community survey, combined with (b) using verbal autopsies, whereby the interpretation of death is determined by a thorough, standardized method that uses a combination of different data sources.

**Table 1:** **Results peer reviewed publications (n = 50)**

**Reference**	**Country**	**Data source**	**Suicide rate**	**Quality score**
Patel et al. (2012) [15]	India	Survey^4^; Autopsy method^9^	22.0	8
Soman et al. (2012) [17]	India	Survey^4^; Autopsy method^9^		6
Bose et al. (2006) [18]	India	Surveillance system^5^; Autopsy method^9^	82.2	6
Abraham et al. (2005) [19]	India	Surveillance system^5^; Autopsy method^9^; Medical records^6^	189.0	6
Prasad et al. (2006) [20]	India	Surveillance system^5^; Autopsy method^9^; Medical records^6^	92.1	6
Aaron et al. (2004) [21]	India	Surveillance system^5^; Autopsy method^9^; Medical records^6^		5
Bose et al. (2009) [22]	India	Surveillance system^5^; Autopsy method^9^	120.3	5
Ahmed et al. (2004) [23]	Bangladesh	Surveillance system^5^; Mortality records^8^; KIIs^11^		5
Hadi (2005) [24]	Bangladesh	Surveillance system^5^; Autopsy method^9^	6.6	5
Khan et al. (2009) [25]	Pakistan	Newspaper reports^10^ *	14.9	5
Sauvaget et al. (2009) [26]	India	Medical records^6^ OR Autopsy method^9^	39.3	4
Gajalakshmi & Peto (2007) [27]	India	Survey^4^; Autopsy method^9^	62.0	4
Kulkarni et al. (2010) [28]	India	Survey^4^; Autopsy method^9^		4
Joseph et al. (2003) [29]	India	Autopsy method^9^	90.9	4
Wasserman et al. (2005) [30]	Sri Lanka	Police records^7^		4
Kavita et al. (2011) [31]	India	Surveillance system^5^; Police records^7^; KIIs^11^		3
Mohanty et al. (2007) [32]	India	Mortality records^8^; KIIs^11^	11.7	3
Abeyasinghe & Gunnel (2008) [33]	Sri Lanka	Autopsy method^9^		3
Yusuf et al. (2007) [34]	Bangladesh	Medical records^6^		3
Feroz et al. (2012) [35]	Bangladesh	Survey^4^	128.8	3
Khan et al. (2008) [36]	Pakistan	Combined^12^	2.9	3
Mayer & Ziaian, (2002) [37]	India	Government crime statistics^1^		2
Girdhar et al. (2003) [38]	India	Government crime statistics^1^	11.2	2
Ambade et al. (2007) [39]	India	Autopsy method^9^; Police records^7^	23.1	2
Mayer & Ziaian (2002) [40]	India	Government crime statistics^1^		2
Steen & Mayer (2004) [41]	India	Government crime statistics^1^		2
Hanwella, & Senanayake (2013) [42]	Sri Lanka	Police records^7^		2
de Silva et al. (2012) [43]	Sri Lanka	Police records^7^; Government health statistics^2^	19.6	2
Thalagala (2009) [44]	Sri Lanka	Police records^7^	24.1	2
Eddleston et al. (2006) [45]	Sri Lanka	Medical records^6^		2
Islam et al. (2002); Islam & Islam (2003) [46,47]	Bangladesh	Mortality records^8^		2
Rahim & Das (2009) [48]	Bangladesh	Mortality records^8^		2
Khan & Hyder (2006) [49]	Pakistan	Police records^7^	1.2	2
Saeed et al. (2002) [50]	Pakistan	Mortality records^8^, KIIs^11^, Police records^7^	1.1	2
Babu & Babu (2011) [51]	India	Government crime statistics^1^		1
Batra (2002) [52]	India	Mortality records^8^; Police records^7^		1
Mohanty et al. (2005) [53]	India	Mortality records^8^; Medical records^6^		1
Steen & Mayer (2003) [54]	India	Police records^7^		1
Sharma (2006) [55]	Nepal	Government census statistics^3^	7.0	1
Khan & Hossain (2011) [56]	Bangladesh	Mortality records^8^		1
ICDDR,B (2003) [57]	Bangladesh	Mortality records^8^	39.6	1
Hossain et al. (2011) [58]	Bangladesh	Mortality records^8^		1
Kanchan et al. (2009) [59]	India	Police records^7^; Mortality records^8^		0
Singh et al. (2003) [60]	India	Mortality records^8^		0
Singh et al. (2005) [61]	India	Mortality records^8^		0
Agnihotram (2004) [62]	India	Mortality records^8^; Government census statistics^3^; Survey^4^		0
Sharma et al. (2006) [63]	Nepal	Mortality records^8^		0
Fernando et al. (2010) [64]	Sri Lanka	Police records^7^		0
Hoq et al. (2010) [65]	Bangladesh	Mortality records^8^		0
				Mean 2.6

Note: *Verified with a standardized questionnaire for police, health personnel, and religious leaders; ^1^National Crime Records Bureau data (Ministry of Home Affairs); ^2^Ministry of Health data; ^3^National census data.

^4^National and Sub-national (community, household, or rural) surveys; ^5^Computerized surveillance systems for subnational populations; recording health, demographics, or injuries; ^6^Hospital or medical records.

^7^Police records and inquest reports; ^8^Official records of death: Mortuary data, death registration forms, medical/medico-legal autopsy reports; ^9^Primary autopsy data: Verbal or psychological autopsy data collected for the study.

^10^National and local newspaper reports; ^11^Primary interview data from health staff, police, family or acquaintances of the deceased; ^12^Secondary data from published sources.

Suicide data in South Asia show that overall more men commit suicide than women. According to WHO data the ratio in the Southeast Asia region (which includes Bangladesh, India, Nepal and Sri Lanka) was 1.57: 1 [male : female], and in the Eastern Mediterranean Region (which includes Afghanistan and Pakistan), was 1.42: 1 [66]. The findings from this scoping review generally correspond to these ratios, except for studies from Sri Lanka and Bangladesh. Sri Lanka reported very high male rates compared to female rates (3.11:1 to 3.79:1), while Bangladesh reported inverse male to female ratios, i.e. more female suicide deaths than male (0.43:1 to 0.83:1). (See Additional file 2.) In addition, younger women of reproductive age seem to be at highest risk among females, and it is the only age group where women’s rates meet or exceed male rates, across the South Asian countries where information is available [15,55]. In the 15-29 year age group several sources including the latest WHO statistics from the region demonstrate suicide to be (among) the leading cause(s) of death [1]. See Additional file 3. While overall reported suicides are higher amongst men, the only gender-specific studies we found focused exclusively on women (n = 10).

Less than half of the included studies (48/114, 42.2%) contained disaggregated information on multiple means of committing suicide. Poisoning and hanging are the two leading means of committing suicide across the sub-continent, with context-specific variations. There is no common definition of suicide in the literature reviewed. The majority (41/49 or 84%) of published studies did not explicitly define suicide in the context of their study. An even larger proportion of the grey literature (59/64 or 92%) did not provide definitions of suicide.

## Discussion

A scoping review is a specific type of review, which can provide a structured approach to mapping available information on a specific subject. Scoping reviews differ from other types of systematic reviews in that they provide a broad map of the existing literature without meta-analyses of the data. Scoping reviews can be used to inform a future systematic review, but also to explore the extent of the literature on a certain topic, including research findings and gaps [7,9]. The results of this review provide an overview of the information that is available in the literature about suicide deaths in South Asia.

Compared to the most recent global average suicide rates (11.5) [1-3], the average rate for the six south Asian countries is clearly higher when including more conservative nationally representative studies only, and much higher when including all available reports including data on specific sub-populations. The problem of suicide is generally more pronounced among men, and particularly severe among women in the 15-29 year age group, where several sources find it to be the leading cause of death. Rates are especially high in Bangladesh, India and Sri Lanka. However, comparisons between and within countries based on mean rates are problematic, because of the differences in data methods (for example some rates are age-adjusted, whereas most are not) and validity of data. For example, there are large differences between reported rates in national and sub-population studies. Possible explanations for high rates among the sub-populations include study bias, i.e. the sub-population studies may have represented a population at increased risk, and divergent methods of establishing suicide rates. The national data is mainly gathered through suicide or mortality registration systems (and in a few cases through large national mortality surveys), with police often as the primary repository and source of data (suicide is considered a criminal offence in five of the six countries), which may explain the lower rates. Only India and Sri Lanka publish official annual national suicide data. Pakistan, Afghanistan, Bangladesh and Nepal (together representing only 12% of the studies included in this review) have no systematic suicide surveillance system, and rely mostly on police data which are likely gross underestimations of actual rates. The study of Patel and colleagues [15] in India, the only study in the data set that scored positive on all quality indicators, is an important case in point. It demonstrates that a nationally representative cause of death survey results in significantly higher reported suicide rates compared to the National Crime Records Bureau – the most commonly used reference for suicide rates in India. Afghanistan is notable for its almost complete absence in the report. Although 32 documents were collected, only one document was included in the final review, and that focused on terrorist suicide attacks.

The paucity of official statistics and data is perhaps unsurprising, given the lack of resources and funding for research, and the competing health and development priorities within these low- and middle income South Asian countries [67]. The impact of suicide being a criminalized act in all countries except Sri Lanka, is also bound to limit the accuracy of information about suicidal acts, particularly as police records are the main source of available data.

Quality of studies reporting on suicide rates is generally low, with only 10 of 50 scientific publications meeting more than half of the quality criteria. The omission of a definition of suicide in most of the publications is an evident example of issues with reliability within this study set. Higher quality studies in this review generally combined several data sources including data from large representative samples (i.e. national health/mortality surveillance system or community survey) and routine standardized verbal autopsies, guided by a clear definitions or classification system, (i.e. validity) and are adequately analyzed and presented (i.e. adjusting for age, including confidence intervals).

A strength of this review is the broad approach followed inherent to a scoping review methodology: combining a systematic review of the published literature with in-country searches, providing a more comprehensive overview. By comparison, another review of suicide in Asia only has nine publications relevant to the six south Asian countries studied in the current review [4]. This strategy is especially suitable for problems with scarce available data, such as suicide rates, and may well be useful for other parts of the world. The review also had several limitations. First, we used a novel approach to rate quality of studies. While we did assess IRR, which showed good to very good reliability between different researchers using the instrument, other psychometric properties of the tool developed for this study have not been evaluated. Second, the use of national consultants to conduct the in-country search for reports and data may have introduced some bias as it was difficult to fully standardize this component between countries. Third, in the results section we have reported mean suicide rates. As mentioned before, taking a mean of such varying data is potentially problematic. An actual arithmetic mean score could not be calculated because many of the publications and reports did not report absolute suicide and population numbers. Restricting the review to a specified time-period, which was done to focus on current trends, can be considered a further limitation.

Nevertheless, our findings have important research and policy implications. First, there is a critical need to establish national suicide surveillance systems in the South Asian countries where they currently do not exist, and to evaluate the reliability of the systems that are in place in India and Sri Lanka. In the absence of data collection systems, high quality nationally representative cause of death studies can play an important role in getting a better picture of the real magnitude of the problem. Second, overall the reported suicide rates in South Asia are high compared to the global average, especially considering that the problems with validity and reliability will more likely obscure rather than exaggerate the magnitude of suicide deaths. This calls for increased public health attention and comprehensive suicide prevention programs. Third, a research agenda needs to be formulated to address the gaps in the current knowledge base, which should include replicating high quality studies such as the one by Patel and colleagues in India [15]. It is equally urgent to gain in-depth understanding of other aspects of suicide in the region, including self-harm and suicidal ideation, risk and protective factors, and existing prevention efforts, so that an adequate response can be designed and implemented.

## Conclusion

The reported suicide rates in South Asia are high compared to the global average, but there is a paucity of reliable data on suicide rates in South Asia, especially national level and high quality data. Reports are likely to obscure rather than exaggerate the magnitude of suicide rates due to lack of quality- and nationally representative data, as well as the reliance on reporting by police in most of the settings. Study population and methods of data collection are key predictors of reported suicide rates: higher quality studies consistently report higher rates than lower quality studies, and sub-population studies report higher rates than national level data. There is an urgent need to get more reliable suicide data. This can be done through establishing new, or evaluating existing, national suicide surveillance systems in the South Asian countries. It can also be achieved by studies that combine several data sources, including data from large representative samples (i.e. surveillance systems) and routine standardized verbal autopsies, guided by a clear definition or classification system, and that analyze data, adjusting for age, with accurate presentation of data including confidence intervals. Further investigation is urgently needed to ensure that public health policy and interventions are put in place.

## Additional files

Additional file 1:
**Overview of all studies and reports included in the review (n = 114).** This table provides an overview of key characteristic and findings of all studies included in the review, both grey literature and peer reviewed publications. Additional file 2:
**Male to female ratios of suicide rates.** This table presents suicide rates by gender, for the publication for which this information was available. Additional file 3:
**Age disaggregated data.** This table provides age disaggregated data on suicide rates, for the publication for which this information was available.
