# Supplementary material for: Suicide in South Asia: a scoping review
Source: BMC Psychiatry. 2014 Dec 24;14:358. doi: 10.1186/s12888-014-0358-9 (PMC4299381; doi:10.1186/s12888-014-0358-9)
Supplement: Additional file 2: — Male to female ratios of suicide rates. This table presents suicide rates by gender, for the publication for which this information was available. [file 12888_2014_358_MOESM2_ESM.pdf]

Additional file 3: Male to female ratios of suicide rates

	**Sri Lanka (Male : Female)**		**India (Male : Female)**		**Pakistan (Male : Female)**		**Nepal (Male : Female)**		**Bangladesh (Male : Female)**
	National	Sub-population	National	Sub-population	National	Sub-population	National	Sub-population	Sub-population
**2011**	3.54 :1*<br>3.77: 1		1.84 :1*		2.0 :1*		1.02 :1*		
**2010**	3.11 :1*		1.86 :1*<br>1.46 :1<br>1.50 :1†		2.34:1*		1.14 :1*		0.43 :1
**2009**	3.36 :1*		1.78 :1*<br>1.67 :1		2.26 :1*		1.21 :1*		
**2008**	3.79 :1 *		1.78 :1*						
**2007**	3.48 :1 *		1.86 :1*	1.19 :1	1.98 :1*				
**2006**	3.76 :1 *		1.78 :1*		1.87 :1*	16 :1			
**2005**	3:59 :1*<br>3.69: 1<br>3.67: 1		1.78 :1*<br>1.67: 1		1.99 :1*				
**2004**			1.78 :1*		0.37 :1*	2 :1			
**2003**			1.70 :1*						
**2002**			1.70 :1*<br>1.20: 1	2.96 :1					
**2001**			1.47: 1				1.60: 1<br>1.25: 1†		
**2000**	2.99: 1<br>2.98: 1		1.47: 1<br>1.45: 1†						
**1994-2010 #**		0.22 :1<br>1.73 :1		1.7 :1<br>5.0 :1<br>1.51 :1		2.3 :1<br>1.6 :1			0.83 :1

Note: Some ratios are calculated by the research team based on presentation of male and female suicide rates; † based age-adjusted rates; * official government rates; # average ratios over multiple years within this period.
