# Supplementary material for: Suicide in South Asia: a scoping review
Source: BMC Psychiatry. 2014 Dec 24;14:358. doi: 10.1186/s12888-014-0358-9 (PMC4299381; doi:10.1186/s12888-014-0358-9)
Supplement: Additional file 3: — Age disaggregated data. This table provides age disaggregated data on suicide rates, for the publication for which this information was available. [file 12888_2014_358_MOESM3_ESM.pdf]

Additional file 2: Age disaggregated data

**Country**	**Year**	**National**	**0-14**			**15-29**			**30-44**			**45-59**			**60+**		
			M	F	All	M	F	All	M	F	All	M	F	All	M	F	All
**India**	2001-2003	Yes	1.2	1.7		25.6	24.9		27.4	15.9		26.2	8.4		27.0	11.2	
	2006-2007	No				82.7	148.5								302.4		
	2002-2007	No											12.6[2]			132.6[3]	
	2004	No					53.7			14.1							
**Nepal**	2008	No		-			31.3			22.8			32.3				
			10-19			20-29			30-39			40-49			50+		
**Sri Lanka**			M	F	All	M	F	All	M	F	All	M	F	All	M	F	All
	2011	Yes	12.0	11.9		14.0	31.9		9.2	32.2		5.3	44.8		6.7	48.6	
	2005	Yes	13.2	13.5		43.4	20.0		49.0	11.4		64.1	8.0		63.0	9.1	
	2000	Yes	20.4	15.32		42.9	20.8		57.0	17.8		67.2	14.0		79.1	17.8	
	2001	Yes						42.0									
**Bangladesh**	1983-2002	No			61.0			42.0			24.5			21.7[1]			

Note: [1] 40+; [2] 45-54; [3] 75+
