# Supplementary material for: Suicide in South Asia: a scoping review
Source: BMC Psychiatry. 2014 Dec 24;14:358. doi: 10.1186/s12888-014-0358-9 (PMC4299381; doi:10.1186/s12888-014-0358-9)
Supplement: Additional file 1: — Overview of all studies and reports included in the review (n = 114). This table provides an overview of key characteristic and findings of all studies included in the review, both grey literature and peer reviewed publications. [file 12888_2014_358_MOESM1_ESM.pdf]

Additional file 1: Overview of all studies and reports included in the review (n=114)

Country	Title	National Yes/No	Population	Years of data (only data from 1998 and beyond are included)	Rates (only data from 1998 and beyond are included [*])	Other suicide metrics	Data source	Quality	Suicide means (top 3 in order of frequency)
India	Suicide mortality in India: a nationally representative survey	Y	Whole India age adjusted for 15 yrs+	2010 estimates and 2001-2003 RHIME survey	22.0*	Suicide= 3% of surveyed deaths (15 yrs or older).<br>At ages 15–29 years, suicide =2nd leading cause of death in both sexes.<br>M:F ratio 1.5: 1<br>M: 26.3, F: 17.6	Nationally representative mortality survey; verbal autopsy (RHIME method)	8	Poisoning: 49% men; 44% women<br>Hanging: 35% men; 26% women
	Burden, characteristics & outcome of injury among females	N	Female population Bengaluru city	2007-8	-	Suicide= 48% of fatal injuries	ISS; Police records and family interviews	3	Hanging: 59%<br>Burns: 22%<br>Poisoning: 15%
	Self-harm & self-poisoning in southern India	N	Kaniyambadi Block, Vellore District, Tamil Nadu	2006-7	120.3*	M:F ratio=1:19:1[a]<br>M rate: 130.9; F: 109.7	CBSS; Verbal autopsy method	5	Poisoning: 43.7%<br>Hanging: 42.5%<br>Burning: 7.9%
	Suicide in South India: a community-based study	N	7 rural villages in Kerala	2002-7	-	Suicide= 6.6% of all deaths<br>M:F ratio= 1.7:1<br>M: 44.7, F: 26.8	Community questionnaire; based on WHO verbal autopsy method and hospital records	6	Hanging: 64%<br>Poisoning: 10%<br>Drowning: 9.3%
	Dowry deaths: a neglected public health issue	Y	Whole India	2005-6	-	31% increase in dowry-related suicides	NCRB	1	-
	Cause of death among WRA	N	WRA in Maharashtra	2003-5	-	Suicide = 35% of injury/poisoning deaths (leading cause of injury death) and 7.2% of total deaths studied.	Household survey; verbal autopsy method	4	Poisoning
	Mortality rate &	N	Rural and peri-	1998-2004	82.2*	Suicide= 11.3% of all deaths	CBSS; Verbal	6	Hanging

	years of life lost from unintentional injury & suicide		urban population Kaniyambadi block, Vellore, Tamil Nadu				autopsy method		Poisoning Burning
	Completed suicide in adults of rural Kerala: rates & determinants	N	Persons 35 years and above Thiruvanantha -puram district	1996-2004	39.3*	Suicide= 3.3% of all deaths studied<br>M:F ratio=5:1<br>M: 78, F:16.5	Medical records or verbal autopsy method (where records were unavailable)	4	Hanging 50%<br>Poisoning 30%<br>Drowning 9%
	Methods of choice in completed suicides: gender differences & review of lit.	N	Population Mangalore (Region district hospital)	2000-3	-	M:F ratio=1.9:1	Police investigation records and medico-legal autopsy records	0	Hanging 36.9%<br>Poisoning 34.7%
	Suicide in India: a four yr retro-spective study	N	Berhampur city and adjacent rural areas	2000-3	11.76*	Suicide= 28% total autopsies<br>M:F ratio= 1.04:1	Medico-legal autopsies; police/ acquaintance interviews of autopsied cases	3	Hanging: 32.6%<br>Poisoning: 30.6%
	Self-Inflicted Burns Fatalities in Manipal, India	N	Catchment area Kasturba Hospital, Manipal. (Burn victims)	1993-2003	-	M: 20.5%, F: 79.5%	Medical autopsies and hospital case records	1	Self-immolation
	Suicide in the elderly in Kaniyambadi, Tamil Nadu, S. India	N	Population of Kaniyambadi Block aged 55 and over.	1994-2003	189* (annual mean rate)	M:F ratio = 1: 0.66 (1.51:1[a])<br>M:234, F: 147<br><br>137.0 among 55-64 year olds<br>204.0 among 65-74 year olds<br>331.0 among 75 or older	CBSS; Verbal autopsy method and health records	6	Hanging 52%<br>Poisoning 39%
	Rates & factors associated with suicide in Kaniyambadi, Tamil Nadu, S.	N	85 villages Kaniyambadi Block, Vellore	2000-2	92.1* (average)	Suicide= 9.8% total deaths studied<br>M:F ratio= 1:0.66 (1.51:1[a])<br>M: 112, F: 72.2	CBSS; verbal autopsy method and health records	6	Hanging: 49%<br>Poisoning 40.5%

	India								
	Spectrum of unnatural fatalities in Chandigarh, NW India – 25 year auto-psy study	N	Population of Chandigarh zone of NW India	1977-2002	-	M:F ratio=0.75:1[a]<br>M: 2.1, F:2.8/ 100,000<br>Proportion of suicidal deaths out of total unnatural deaths studied increased from 6.5% to 15.7% with a peak of 18.2% in 1992–1997.	Medico-legal autopsy records	0	Burns
	Suicides in Imphal	N	Imphal, India.	1993-2001	-	Suicide= 7.1% of total post mortems studied.<br>M:F ratio= 1.98:1	Medical autopsy records	0	Hanging: 52.03%<br>Poisoning: 23.98%
	Suicides in young people in rural southern India	N	Catchment area of Christian Medical College and Hospital, Vellore, Population aged 10-19 years	1992-2001	-	Suicide leading cause of death in 10–19 yrs, M:F ratio=0.39:1[a]<br>M (avg rate): 58<br>F (avg rate): 148	CBSS; Verbal autopsy method and health records	5	Hanging: 44%<br>Poisoning with insecticide: 40%<br>Self-immolation: 9%
	Burn mortality: trends & socio-cultural determinants in rural India	N	Catchment area of Yavatmal district burn ward.	1997-2001		Suicidal burns= 47.8% of total deaths studied<br>Burn mortality rate: 15.1/100,000	Mortuary data and police records	1	Self-burning
	Suicidal & homicidal deaths	N	Catchment area of Apex Medical Centre, District of Maharashtra	1998-2000	23.1*<br>(per year)	Suicide= 19.5% of medico-legal deaths studied<br>M:F ratio= 1.7:1.	Medical autopsy records and police records	2	Poisoning (42.3%)<br>Burning (21.5%).<br>Hanging (20.4%)
	Suicide in India, 1995-1999	Y	Whole India	1995-9	1999: 11.2*<br>1998:10.8 *<br>1997:10.0 †<br>1996: 9.5†<br>1995: 9.7†	M:F ratio= 5.8:4<br>1989-1999: incidence of suicide rose at compound rate of 4.3	NCRB; MoHA data	2	Poison: 37.20%)<br>Hanging: 25.20%<br>Fire/self-immolation: 11.10%

	Evaluation of suicide rates in rural India using verbal autopsy	N	Population of 85 rural villages of Kaniyambadi region, Tamil Nadu	1994-9	1999:90.9*<br>1998:89.8*<br>1997:103.6<br>1996:83.7<br>1995: 106.3<br>1994:96.7	Suicides = between 8% and 12% of total deaths over the study period	Health worker home visits; verbal autopsy method	4	Poisoning (45%)<br>Hanging: (41%)
	Suicide rates in rural Tamil Nadu, S. India: verbal autopsy of 39000 deaths	N	Villupuram district, Tamil Nadu	1997-8	62.0*	Suicides = 9% of total deaths.<br>M:F ratio= 1: 0.72.<br>M: 71, F: 53	Rural survey: verbal autopsy method	4	Self-poisoning: 50%<br>Hanging: 33% (author calculation)<br>Self-immolation: 12.5% (author calculation)
	Suicide, gender, & age variation in India	Y	Whole India	1991-1997	1997: 10.0 †<br>1996: 9.5†<br>1995: 9.7†<br>1994: 9.9<br>1993: 9.5<br>1992: 9.2<br>1991: 9.2	M:F ratio= 1.32:1	NCRB	2	Poison: 34.8%<br>Hanging:23.4 %<br>Self-immolation: 11.5%
	Patterns of suicide by age & gender in Indian states	Y	14/24 Indian states	1997	10.4	Highest suicide rate= 94.3 (males in Kerala)<br>Lowest rate= 0.60 (males in Manipur)	NCRB	1	-
	Modernization & male-female suicide ratio in India 1967–97	Y	All India	1967-1997	-	Rates by gender:<br>1997: M: 11.5, F: 8;60<br>1987: M: 8.49, F: 6.43<br>1977: M: 7.23, F: 5.39<br>1967: M: 8.59, F: 6.53	NCRB	2	-
	Indian suicide & marriage: a research note	Y	All India, age 15+	1995	15.66	M:F ratio=: 1.32:1	NCRB	2	-

	Reviewing disease burden among rural Indian women	N	Female participants of Survey of Causes of Death (rural) 1994 (region unclear)	1994		Female rates by age:<br>15-19: 87.0<br>20-24: 46.3<br>25-29: 27.7<br>30-34: 25.5<br>35-39: 13.4<br>40-44: 3.5	Registrar General of India; annual rural community survey reports; National family health survey	0	-
	Kerala State Mental Health authority website	N	Kerala	2010-11	2011: 25.3*<br>2010: 24.8*<br>2009: 25.5*<br>2008: 25.2*<br>2007: 26.6*<br>2006: 27.0*<br>2005: 27.9*<br>2004: 27.7*<br>2003: 29.6*<br>2002: 30.8*<br>2001: 30.1*<br>2000: 28.8*<br>1999: 30.6*<br>1998: 29.4*	M:F ratio= 3:1	Kerala state MH authority website		Hanging: 57%<br>Poisoning: 26%
	Accidental Deaths & Suicides in India, 2011	Y	All India	2001-11	2011:11.2*†<br>2010:11.4*†<br>2009:10.9*†<br>2008:10.8*†<br>2007:10.8*†<br>2001:10.6*†	M:F ratio: 64.8:35.2 (1.84:1[a])	The Registrar General of India (original source document for official national data)		-
	Accidental Deaths & Suicides in India, 2011	Y	All India	2011	11.2*†		The Registrar General of India		-
	Accidental Deaths & Suicides in India, 2011	Y	All India	2010-11	-	Highest suicide increases: Nagaland (175%), Meghalaya (41.7%) Uttarpradesh (33.5%)	The Registrar General of India		-
	Accidental Deaths & Suicides in India, 2011	Y	All India	2011	-	-	The Registrar General of India		Hanging 33.2%, Poison: 32%; Self-immolation:

									8.8%
	Accidental Deaths & Suicides in India, 2002	Y	All India	2002	2002:10.5*<br>2001:10.6*†<br>2000:10.8*<br>1999 11:2*†<br>1998:10.8*†<br>1992: 9.2	M:F ratio: 63:37 (1.7:1)	The Registrar General of India (based on NCRB data)		
	Accidental Deaths & Suicides in India, 2003	Y	All India	2003	2003:10.4 *	M:F: 63:37 (1.7:1)	The Registrar General of India (based on NCRB data)		
	Accidental Deaths & Suicides in India, 2004	Y	All India	2004	2004:10.5*	M:F: 64:36 (1.78:1)	The Registrar General of India (based on NCRB data)		
	Accidental Deaths & Suicides in India, 2005	Y	All India	2005	2005:10.3*	M:F: 64:36 (1.78:1)	The Registrar General of India (based on NCRB data)		
	Accidental Deaths & Suicides in India, 2006	Y	All India	2006	2006:10.5*	64:36 (1.78:1)	The Registrar General of India (based on NCRB data)		
	Accidental Deaths & Suicides in India, 2007	Y	All India	2007	2007:10.8*†	65:35 (1.86:1)	The Registrar General of India (based on NCRB data)		
	Accidental Deaths & Suicides in India, 2008	Y	All India	2008	2008:10.8*†	64:36 (1.78:1)	The Registrar General of India (based on NCRB data)		
	Accidental Deaths & Suicides in India, 2009	Y	All India	2009	2009:10.9*†	64:36 (1.78:1)	The Registrar General of India (based on NCRB data)		
	Accidental Deaths & Suicides in India, 2010	Y	All India	2010	2010:11.4*†	M:F: 65:35 (1.86:1)	The Registrar General of India (based on NCRB data)		2010: Poisoning 33.1% Hanging 31.4% Self-

									immolation 8.8% (BUT in cities, hanging leads)
	Suicide rates, India, WHO	Y	All India	1980-2009	2009: 10.5*<br>2005: 9.9*<br>2000: 10.6*<br>1995: 9.6<br>1990: 8.9<br>1985: 7.1<br>1980: 6.3	2009: M:13.0 , F:7.8<br>2005: M:12.3, F:7.4<br>2000: M:12.6 , F:8.6<br>1995: M:10.9, F: 8.2<br>1990 M:10.5, F: 7.3<br>1985: M:8.2 , F: 6.0<br>1980: M: 7.3, F:5.3	NCRB; MoHA (Via WHO)		
	Report on Causes of Death in India, 2001-03	Y	All India	2001-3		Intentional self -harm (suicide)= leading cause of death in 15-24 (15.6%) and 25-34 (10.2%) age groups	Survey of Deaths under SRS; Verbal autopsy method (RHIME method)		-
	Study of domestic burns in young women, from Mental Health Research in India	Y	Female burn victims	1988	-	-	Psychological autopsy		Self-burning
	Suicides in India, Maithri	Y	All India	1989-2011	2011:11.2*†<br>2010:11.4*†<br>1989:8.47	-	NCRB	-	-
	Cause of death among WRA in rural N. India	N	WRA, Rural N India	2006-11	-	Suicide =10.2% of overall deaths	Medical records	-	-
	Suicides in Northern India Comparison of trends & review of lit	N	Punjab, Haryana and Himachal Pradesh	2005	-	Suicides = 44.7% of total unnatural deaths. Increase in the percentage of suicides from 34% in 1996 to 49% in 2005	Hospital and inquest records; then police and family info		Poison= 47% Self-immolation: 39%. Hanging: 8%
	Suicides in Kerala: an analysis	N	Kerala	1995-2002	2002:30.24*†<br>2001:29.74*†<br>2000:29.22*†<br>1999:31.00*†<br>1998:29.79*†<br>1997:28.96	2002:M:F ratio:73: 27<br>Rates:M:38.06, F:15.35<br>2001:M: 42.72, F:16.85<br>2000:M:42.72, F:16.46<br>1999:M:44.72, F:18.04<br>1998:M:42.84, F:17.45	NCRB		

					1996:26,38<br>1995:26.38	1997:M:41.33, F:17.26<br>1996:M:36.35, F:16.95<br>1995:M:38.06, F:15.35			
	Suicide Mortality Rates across States of India, 1975-2001	Y	All India<br>Age adjusted 5 yrs +	1975-2001		2001: M:14.0, F: 9.5<br>2000: M:14.2, F: 9.8<br>1999: M:14.4, F:10.6<br>1998: M:13.8,F:10.4<br>1997: M:12.9, F:9.7<br>1996:M:11.9, F:9.3<br>1995: M:12.5, F9.5<br>1990: M:11.5, F:8.7<br>1985: M:9.0, F:7.1<br>1980: M:8.0, F:6.2<br>1975: M:9.7, F:6.8	NCRB		
Sri Lanka	Age & sex specific suicide rates in Sri Lanka 1995-2011	Y	All Sri Lanka	1995-2011	-	2011 M:F ratio=: 3.76: 1<br>2011: M: 34.8, F: 9.24<br>2005: M: 46.04, F: 12.47<br>2000: M: 51.75, F: 17.3<br>1995: M: 89.94, F:30.79	Suicide data from police records, population data from WB database	2	-
	From pesticides to medicinal drugs: time series analyses of methods of self-harm in Sri Lanka	Y	All Sri Lanka	1995-2009	2009: 19.6*<br>1995: 47.0†	-	Police records and MoH data	2	-Self-Poisoning<br>-Non-poisoning methods.<br>Rates show reduction of 26.2/ 100,000 for poisoning & 1.5 for non-poison-ing between 1995-2009
	Study of suicides reported to the Coroner in Colombo, Sri Lanka	N	Catchment area Colombo Coroners Court	2006	-	-	Coroners court inquest verdicts	0	Self-poisoning: 44%<br>Self-immolation: 34% Hanging 11%
	Suicide trends	Y	All Sri Lanka	1880-2006	2005: 24.1*	2005:M:F 3.7;M:38.2, F:10.4	Police data	2	-

	in Sri Lanka 1880-2006				2000: 28.0*†<br>1995: 47.0†<br>1990: 38.5<br>1985: 38.7<br>1980: 34.8<br>1975:17.7<br>1970: 19.2<br>1965:13.7<br>1960: 9.9<br>1880: 2.3	2000 M:F 3.0;M:44.1,F:14.8<br>1995:M:F 2.7;M:68.3,F:25.2<br>1990:M:F 2.9;M:56.8,F:19.5<br>1985:M:F 2.2;M:48.8,F:22.3<br>1980:M:F 2.1:M:46.7,F:22.1<br>1975:M:F 2.2;M:24.0,F:11.1<br>1970:M:F 2.3;M:26.3,F:11.5<br>1965:M:F 2.3;M:18.7,F: 8.2<br>1960:M:F 2.0;M:12.7,F: 6.3			
	Patterns of hospital transfer for self-poisoned patients in rural Sri Lanka	N	Population Anuradhapura district	2002	27.0<br>(for fatal self-poisoning)	-	Medical records	2	Self-poisoning (only method studied)
	Psychological autopsy study of suicide in three rural & semi-rural districts of Sri Lanka	N	Population 3 rural districts: Kurunegala Hambantota Anuradhapura	1997	71.0<br>(average)	-	Community based psychological autopsies	3	Self-poisoning: M=70.0%; F=68.4%<br>Hanging: M= 12.6; F= 3.8%
	Global suicide rates among young people aged 15-19	N	15-19 year olds globally	1980-1986 (SR data)	1986: 46.5	M: 43.9; F: 49.3	WHO mortality database 2004	4	-
	Crime trends, Sri Lanka Police Website	Y	All Sri Lanka	2005-2011	--	Absolute numbers<br>2011: M:F ratio: 2939:831 (3.54:1[a])<br>2010 (1/2 yr only): M:F: 1531:492 (3.11:1)<br>2009: M:F: 3097:921 (3.36:1)<br>2008: M:F: 3260:860 (3.79:1)<br>2007: M:F: 3281:944 (3.48:1)<br>2006: M:F: 3558:946 (3.76:1)	Police		Pesticides: M:1447/ 2939<br>F: 310/831<br>Hanging: M:1122/ 2939<br>F: 224/834

						2005: M:F: 3708:1034 (3.59:1			
	Suicide rates by gender and age, WHO website	Y	All Sri Lanka	1996	1996: 21.6<br>1991:31.0<br>1985: 35.8<br>1980: 29<br>1967: 16.9<br>1960: 9.9<br>1955: 7.4<br>1950: 6.5	--<br>M: 44.6, F: 16.8<br>M: 48.8, F: 22.3<br>M: 37.3, F: 19.7<br>M: 23.3, F: 10.0<br>M. 13.2, F: 6.0<br>M: 9.8, F: 4.8<br>M: 9.6, F: 3.9	WHO Website		
	Sri Lanka Sumithrayo Rural Program Annual Report	N	80 of most suicide-prone villages in the Northwestern and Southern provinces	2009-10	2010: 37.6*<br>2009: 32.98*<br>2008: 26.9*<br>2007: 38.0*<br>2006: 32.8*<br>2005: 37.78*<br>2004: 29.23*<br>2003: 41.8*		Police records		Poisoning<br>Hanging
	Suicide in Sri Lanka: Past, Present & Future Transformations	N	WRA	2002-10	-	12.1/100,000 live births	Maternal Mortality Surveillance system		Poisoning: 38%<br>Burns: 23%<br>Hanging: 16%
	Sumithrayo website	Y	All Sri Lanka	1993-2009	2009:20.3*<br>2008: 20.4*<br>2007: 21.1*<br>2006: 22.7*<br>2005:23.2*<br>2004: 24.2*<br>2003:23.1*<br>2002:23.8*<br>2001:25.4*<br>2000:28.0*<br>1999:31*<br>1998:31.1*<br>1997:34.6<br>1996:40.1<br>1995:47<br>1994:43.1<br>1993:41.7		Census and Statistics Dept; Police Dept Suicide figures		

	Suicides among children in Monaragala District in Sri Lanka	N	Monaragala District; Children aged 15-19	2000-6		Wellawaya<br>2005, M: 35, F: 24<br>2004, --<br>2003, M: 12, F: 12<br>2002, M: 12, F: 12<br>2001, M:24, F: --<br>Thanamalwila<br>2006, M: --, F: 50<br>2005, M: --, F: 25<br>2004, M: --, F: 51<br>2003, M: --, F: 26<br>2002, M:25, F: 52<br>2001-2000: M: 26, F: 27	Local police data, Official documents; questionnaire survey; interviews		Ingestion of pesticide 80-82%
	Suicide report from Psycho-social Forum of Consortium of Humani-tarian Assn	N	Population Trincomalee	2004-5	27.4*		Hospital and police records		Men: poisoning or hanging<br>Women: burning
	National Report on Violence & Health<br>Sri Lanka	Y	All Sri Lanka	1880-2004	2001: 26.5*	2004: M:F ratio: 3.5:1	Department of Health Services. Annual Health Bulletin 2002		2004<br>Poison: 63.4%
	Suicide among women in Sri Lanka	N	Catchment area Coroners' Courts Colombo	2000-4	-	Suicides= 4-6% of sudden deaths<br>M:F ratio = 1.6:1	Coroner's records of sudden deaths		Men: self-poisoning<br>Women: self-immolation
	Intention-al self-poisoning<br>in rural Sri Lanka	N	Population of rural area, southern Sri Lanka	1990-2002	-	Median incidence rate for self-poisoning = 158<br>Rate refers to all poisoning (including fatal)	Recorded hospital admissions		Self-poisoning (only method studied)
	Youth suicides in Sri Lanka	N	Youth of Anuradhapura, Kurunegala and Hambantota, districts	Aug-Oct, 1997	104	M:F ratio: 1.28	Police, hospital, coroner, Registrar, Samurdhi Agents.		Pesticides: 76%
Banglad esh	Community survey onsuicidal	N	Population Sadar Upazilla of district	2009-10	128.8*	M: 77.9, F: 183.1	Community-based survey	3	Common methods (NO ORDER):

	attempts & deaths in rural area of Bangladesh		Chuadanga						hanging, ingestion of insecticides, burning, jumping in front of train
	Suicidal death autopsy analysis at Dhaka Medical College	N	Catchment area Forensic Dept, Dhaka Medical College	2008-9	-	Suicide= 19% total deaths studied<br>Female: 60.1% Male: 39.9%	Medical autopsy reports	1	Hanging 59%<br>Poisoning: 31%
	Manner of deaths at a selected medical college morgue house	N	Catchment area Salimullah medical College, Dhaka	2008	-	Suicide= 20.4 % total deaths studied<br>(2nd leading cause of death)<br>M: 68.75%, F: 31.25%	Medical autopsy reports	0	-
	Mortality due to suicide in rural Bangladesh	N	Population rural and semi-urban sub districts of Jessore District	1983-2002	39.6*	Suicide= 8% total deaths studied (5th most common cause of death)	Autopsy records	1	Poisoning:<br>F =84%,<br>M=72%
	Study on unnatural death patterns in Dhaka City	N	Population Dhaka metro city	1996-2000	-	Suicides= 10.76% total deaths studied<br>M: 26.55%, F: 73.45%	Medical autopsy reports	1	-
	Risk factors of violent death in rural Bangladesh, 1990-1999	N	70 Villages covered by demographic surveillance system (DSS)	1990-9	6.6*	Suicide: 3rd main cause of violent deaths (: 23.43%)*<br>Suicide deaths higher among women than men :M:F= 0.47:1*	DSS; verbal autopsy method	5	-
	Violent deaths among WRAin rural Bangladesh	N	Bangladesh WRA	1982-1998	-	Suicide rates per 10,000 person years:<br>Males: 0.8<br>Females: 1.3<br>5.48% suicide of total deaths (author calculation)	Longitudinal DSS; Death registration forms (verified by family interviews)	5	-
	A ten year retrospective	N	Catchment area of Dhaka	1988-1997	-	Suicides= 18.43% total post-mortem cases	Autopsy reports	2	Poisons: 58.46%

	study of suicide cases reported at Sir Salimullah Medical College Mortuary		mortuary			M: 54.18%, F: 45.82			Hanging: 40.90%. (nb: only 178/ 467 means were documented)
	A ten year retrospective study of suicide cases reported at Sir Salimullah Medical College Mortuary (Same study as above but separate publication)	N	Catchment area of Dhaka mortuary	1988-1997	-	See above	Autopsy reports	2	
	Causes of death among women aged 10-50 years in Bangladesh	Y	Women aged 10-50	1996-7	9.0	Suicide= 10.7% all deaths studied, 47% of injury deaths studied (Suicide= 3rd leading single cause of death overall, & single leading cause of death among 15-19 yr olds	Case reports from medical records and health service staff interviews	3	-
	Mortuary profile for unnatural deaths at Forensic Med Dept, Dhaka Medical College	N	Catchment area Dhaka Medical College, Dhaka city	1996	-	Suicide accounted for 8% unnatural deaths. M:F= 1.7:1	Medical autopsy reports	2	Poisoning Hanging
	Mortality Profile Bangladesh 2011	N	Catchment area of 451 govt health facilities	2011	-	Suicides = .09% of all deaths reported	Govt. health facility reports		-
	Record of unnatural deaths in Bangladesh, 2003-2010	Y	All Bangladesh	2003-10	-		Police headquarters' records		Poisoning (absolute figures show an increase over study

									period)
	Suicide report of 6 sub-districts of Jheneidah	N	Population Jheneidah district	2003-2012	-	Annual absolute numbers of attempts, deaths, disaggregated by sex and means	Police (district and subdistrict) and hospital records		Hanging Poisoning/ sleeping pills
	Suicide report of 6 subdistricts of Jheneidah	N	Population Jheneidah district	2011	-	Monthly absolute numbers of attempts, deaths, disaggregated by sex and means	Police and hospital records		Poisoning/ sleeping pills
	Health & demographic surveillance system (HDSS) Matlab Registration of health & demographic events 2010	N	Population of Matlab	2010	-	Rates /1000:<br>Service group:<br>M: 11.07<br>F: (6.61)<br>Control:<br>M: (9.49)<br>F: 10.20	DSS		-
	HDSS, Matlab Registration of health & demographic events 2009	N	Population of Matlab	2009	-	Rates /1000:<br>Service group:<br>M: (4.71)<br>F: 12.81<br>Control:<br>M: (1.64)<br>F: 9.75	DSS		-
	HDSS, Matlab Registration of health & demographic events 2008	N	Population of Matlab	2008	-	Rates /1000:<br>Service group:<br>M: (1.47)<br>F: (4.65)<br>Control:<br>M: 8.42<br>F: (6.97)	DSS		-
	HDSS, Matlab Registration of health & demographic events 2007	N	Population of Matlab	2007	-	Rates /100,000:<br>Service group:<br>M: 0<br>F: (4.76)<br>Control:<br>M: 11.05	DSS		-

						F: 9.59			
	HDSS, Matlab Registration of health & demographic events 2006	N	Population of Matlab	2006	-	Rates /1000:<br>Service group:<br>M: (4.60)<br>F: (1.48)<br>Control:<br>M: (7.41)<br>F: 14.28	DSS		-
	HDSS, Matlab Registration of health & demographic events 2005	N	Population of Matlab	2005	-	Rates /100,000:<br>Service group:<br>M: (1.56)<br>F: 13.28<br>Control:<br>M: (3.86)<br>F: (4.75)	DSS		-
	HDSS, Matlab Registration of health & demographic events 2004	N	Population of Matlab	2004	-	Rates /100,000:<br>Service group:<br>M: (4.43)<br>F: 0<br>Control:<br>M: 9.54<br>F: 9.61	DSS		-
	HDSS, Matlab Registration of health & demographic events 2003	N	Population of Matlab	2003	-	Rates /100,000:<br>Service group:<br>M: 0<br>F: (2.68)<br>Control:<br>M: (2.57)<br>F: (1.63)	DSS		-
	HDSS, Matlab Registration of health & demographic events 2002	N	Population of Matlab	2002	-	Rates /100,000:<br>Service group:<br>M: (5.72)<br>F: 9.93<br>Control:<br>M: (4.97)<br>F: (1.39)	DSS		-
	HDSS, Matlab Registration of health & demographic	N	Population of Matlab	2001	-	Rates /100,000:<br>Service group:<br>M: 2.75<br>F: 2.82	DSS		-

	events 2001					Control:<br>M: 11.17<br>F: 1.66			
	Mortality due to suicide & homicide in rural Bangladesh	N	Married WRA	1983-2002	-	Suicide and homicide=5% of all deaths<br>(90% suicide, 10% homicide)<br>Deaths due to suicide & homicide higher among females (65%),	DSS: verbal autopsy; household survey		-
Nepal	A review of violent & traumatic deaths Kathmandu, Nepal	N	Catchment area Forensic Medicine dept. Kathmandu	2000-2004		Suicide= 25% total fatalities studied (second leading cause after accidents)	Autopsy records	0	Hanging: 54%<br>Poisoning 14%<br>Burns: 5%<br>Undetermined: 23%
	Leading causes of mortality from diseases & injury in Nepal	Y	All Nepal	2001	7.0*<br>9.0*<br>(15-65 years)	Suicide= 9th leading cause of death for all ages<br>M: 8/100,000<br>M: 10 (15-65 yrs)<br>F: 5/100,000<br>F: 8 (15-65 yrs)	2001 census sample survey	1	-
	Suicide Record 2009/10 to 2011/12	Y	All Nepal	2009-12	-	2011/12: M:F ratio: 1942:1906 (author recalculation: 1.02:1)<br>2010/11: M:F: 1904:1663 (1.14)<br>2009/10: M:F: 1820:1507 (1.21)	Police records		Hanging<br>Poison<br>Self-burning
	Causes of death of foreign workers	N	Nepalese foreign employees	2007-12	-	Absolute numbers of suicides and other deaths	Foreign Employment promotion board reports		-
	Foreign labor migration & trafficking in Nepal	N	Nepalese foreign employees	2007-12	-	Suicide= 9.4% of all deaths of Nepali foreign labor migrants<br>(May 2008 – Sep 2012)	Foreign Employment promotion board reports		
	Epidemiolog-ical study on injury & violence	Y	All Nepal	2007-8	-	NA[b]	Tertiary care center records		-

	in Nepal								
	Nepal Maternal Mortality & Morbidity Study 2008/2009	Y	WRA in all Nepal	2008	-	Suicide = 16% of all deaths<br>Suicide = nearly 50% non-maternal pregnancy related deaths<br>Suicide= single leading cause of death for WRA	The Maternal Death Review (endorsed by WHO); verbal autopsy method		Poison (mainly pesticides): 57%<br>Hanging: 39%
	Legal study of suicide incidents or cases & collaborating factors in Nepal	Y	All Nepal	-	-	NA[b]	Newspaper reports		Hanging: 54%<br>Jumping into rivers/ off buildings:17%<br>Poisonings: 14%
	Assessment of psycho-social needs & suicide risk<br>factors among Bhutanese refugees	N	Bhutanese refugees in Nepal	2004-11	09/10:20.76*		Certified cases from UNHCR database		Hanging (almost all)
	Acid & burns violence<br>in Nepal,<br>a situational analysis	N	Victims and survivors of acid and burn violence	2003-9	-	Rate of suicide increased10% compared to population increase of 3% from 2003/4-2007/8.	Police data/ secondary lit		Self-immolation (only method studied)
	Security & justice<br>in Nepal,<br>district assessment findings	N	Banke, Jumla, Kailali, Nawalparasi, Siraha and Sunsari districts	2008-9		Banke district: suicide= 24.34% of total police cases (73/204) (2nd[d] most common after vandalism)<br>Nawalparasi: attempted suicide= 31.48% of total police cases (85/270) [b]	District Police records		
	A review of the evidence:<br>suicide among women in Nepal	Y	WRAs (15-49) in Nepal	2008	Police data:<br>09/10 = 4.7*<br>08/09 = 2.5*<br>07/08 = 3.0*<br>06/07 = 3.5*<br>05/06 = 4.3*<br>04/05 = 5.8*<br>03/04 = 4.9*		Multiple secondary sources		Police data, 2003-2011:<br>Hanging: 59%<br>Poison: 35%<br><br>MMMS, 2008:<br>WRAs, 15-50:<br>Poisoning:

					MMMS data, WRAs:<br>08/09: 28*<br>1998: 22*				56%<br>Hanging: 41%
Pakistan	Epidemiology of suicide in Pakistan: determining rates<br>in six cities	N	Study populations of systematic review: Larkana, Rawalpindi, Peshawar, Lahore, Faisalabad, Karachi	1993-2006	2006, Rawalpindi: 2.86*<br>2002-4, Larkana: 2.6*<br>1998-2001, Faisalabad: 1.12*<br>1995-2001, Karachi: 2.12*<br>1991-2000, Peshawar: 0.43*<br>1993-1995, Lahore: 1.08	2006<br>M:F ratio: 16:1<br>M: 5.2, F:0.34<br>2002-4, M:F ratio: 2:1<br>M: 3.3, F:1.77<br>1998-2001<br>M:F ratio: 2.3:1<br>M: 1.51, F:0.68<br>1995-2001, M :F ratio: 1.6:1<br>M: 2.49, F:1.70<br>1991-2000<br>M :F ratio: 2.9:1<br>M: 0.61, F: 0.23<br>1993-1995, M :F ratio: 1.8:1<br>M: 1.3, F:0.79	Systematic review: Suicide studies of at least 1 year over last 20 years	3	-
	Female suicide rates in Ghizer, Pakistan	N	Women in Ghizer District	2000-4	14.89*	Women over 15: 33.22<br>Women 15-24: 61.07	National and local newspaper reports (verified with qu'airre for police, health personnel religious leaders etc.)	5	Jumping in river/lake: 40%<br>Poison: 33%<br>Hanging/ Strangula-tion: 11%
	Epidemiology of suicide in Faisalabad	N	Population of Faisalabad	1998-2001	1.12*<br>(average)	M:F Ratio= 2.4: 1<br>Suicide=10.3% total autopsies reviewed	Autopsy reports (subsequent family interviews, police inquests and crime scene visits)	2	Hanging: 51.51%<br>Firearm: 18.17%<br>Poisoning: 15.15%.
	Suicides in the developing world:<br>case study from Pakistan	N	Population of province of Sindh	1985-1999	1.15*	M:F Ratio overall: 2.5	Police records	2	Poisoning: 40%<br>Hanging: 27%<br>Drowning: 17%

	(7 docs)<br>Suicide reports from the Human Rights Commision of Pakistan	Y	All Pakistan	2004-11	-	Absolute numbers of suicides, broken out by sex, means, marital status, etc.<br>2011: M:F ratio:1411:704 (,2.0:1[a])<br>2010: M:F: 1677:718 (2.34:1)<br>2009: M:F: 1270:563 (2.26:1)<br>2007: M:F: 1371:692 (1.98:1)<br>2006: M:F: 1429:766 (1.87:1)<br>2005: M:F: 1037:520 (1.99:1)<br>2004: M:F: 197:527 (0.37:1)	Various secondary sources including press and international agency reports		Poisoning most common by far all years, followed by shooting and/or hanging
Afghani stan	Suicide attacks in Afghanistan (2001-2007)	Y	Afghan suicide attackers	2002-2007	-	-	UNDSS Database system		Body borne and vehicle borne suicide attacks using explosive material
	Suicide prevention in Asia,WHO	Y	All India	2002	17.38*	M:F ratio=1.2:1<br>Male rate: 18<br>Female rate: 15	2002 suicide estimates: WHO (2007)		Poisoning: 38%<br>Hanging: 29%
			All Sri Lanka	2003	23.9*		2003 suicide estimates: National Police Department statistics		Poisoning by pesticides: 40-80%

Note: * Rates are included in quantitative analyses; † Same data presented in multiple studies; [a] These M:F ratios were calculated by the authors based on the male and female suicide rates presented in the report; [b] There is insufficient information to determine whether the reported figures refer to ‘suicide deaths’ or ‘suicide attempts’.

WRA= Women of reproductive age

DSS= Demographic Surveillance System

HDSS= Health and Demographic Surveillance System
